# Death by Neighborhood: Preliminary Results on Disparities in Violent Deaths by Neighborhood Opportunity With the Child Opportunity Index 3.0

**DOI:** 10.1016/j.focus.2025.100357

**Published:** 2025-04-29

**Authors:** Manning Zhang, Clemens Noelke, Dolores Acevedo-Garcia, Robert W. Ressler

**Affiliations:** 1 Department of Sociology, Brandeis University, Waltham, Massachusetts; 2 The Heller School for Social Policy and Management, Brandeis University, Waltham, Massachusetts; 3 School of Social Work, Boston University, Boston, Massachusetts

**Keywords:** Violent death disparities, neighborhood opportunity, health disparities, social determinants of health

## Abstract

**Introduction:**

This study examines inequities in violent death rates among children aged 0–19 years across different levels of neighborhood opportunity.

**Methods:**

The authors combined nationally representative 2020 data from the National Violent Death Reporting System, the Decennial Census, and the Child Opportunity Index 3.0 to estimate death rates by neighborhood opportunity.

**Results:**

The crude violent death rate declines monotonically with neighborhood opportunity from 15.3 deaths per 100,000 children in very low-opportunity to 3.8 per 100,000 children in very high-opportunity neighborhoods. Homicide death rates are 13.3 times higher in very low- (10.5 deaths per 100,000 children) than in very high-opportunity (0.8 deaths per 100,000 children) neighborhoods. There were 3.3 suicide deaths per 100,000 children in very low- compared with 2.7 deaths per 100,000 children in very high-opportunity neighborhoods.

**Conclusions:**

The findings highlight the Child Opportunity Index’s value in analyzing stark geographic disparities in children’s violent death risk. They underscore neighborhood context as a key risk factor and suggest that place-based interventions could help mitigate these disparities. However, neighborhood differences in suicide mortality were minor, warranting further research on the suitability of neighborhood indices for predicting spatial variations in suicide risk.

## HIGHLIGHTS

• In 2020, children’s violent death rates varied by Child Opportunity Index levels.
• The neighborhood opportunity association is stronger for homicides than for suicides.
• The Child Opportunity Index maps conditions associated with geographic disparities in violent deaths.
• Findings support cause-specific place-based violence prevention strategies.
• Violence prevention strategies should improve child neighborhood opportunity.

## Graphical Abstract

**Figure:**
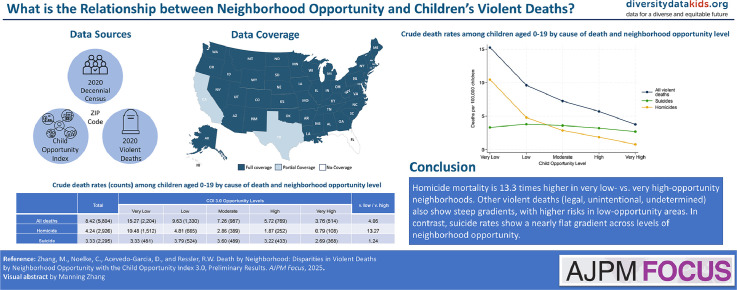

## INTRODUCTION

Geographic disparities in children's violent deaths represent a growing public health crisis, exacerbated by recent trends.^1^, ^2^, ^3^, ^4^, ^5^ However, evidence remains limited regarding the magnitude of these disparities at the neighborhood level.^6^, ^7^, ^8^ This study addresses this gap by leveraging the Child Opportunity Index (COI) 3.0 to quantify neighborhood disparities in children's violent death rates by cause of death in 2020.

The COI is the only composite neighborhood index specifically designed to capture neighborhood characteristics that shape opportunities for children, youth, and families. Unlike the Area Deprivation Index (ADI) and the Social Vulnerability Index (SVI), which exclusively rely on data from the American Community Survey, the COI integrates multiple data sources for a more comprehensive, multidimensional measure of neighborhood opportunity.^9^ It includes neighborhood features such as economic resources, school quality, exposure to pollution, ambient heat, access to safe outdoor spaces, and community social capital, which previous research has linked to mental health, crime, and violent death outcomes.^10^, ^11^, ^12^, ^13^, ^14^, ^15^, ^16^ Compared with the ADI and SVI, the COI captures compounding neighborhood inequities across multiple domains: not only are high-opportunity neighborhoods often wealthier, but they also have access to better jobs, better schools, cleaner air, greener outdoor spaces, and more social resources.^17^ More than 200 health-focused studies have used the COI to examine disparities in children’s health.^18^^,^^19^ Research evaluating its predictive validity suggests that the COI performs as well as or better than other composite neighborhood indices such as the SVI and ADI.^20^, ^21^, ^22^

Despite its increasing prominence as a tool for quantifying health inequities, designing policies, and guiding resource allocation, the COI’s applicability to research and interventions aimed at reducing violent deaths among children remains underexplored. Prior studies linking neighborhood opportunity to violence using the COI have examined only single metropolitan areas or did not differentiate between self-inflicted and assault-related injury or death.^4^^,^^6^^,^^23^^,^^24^ Studies examining the relationship between COI and suicidality have yielded mixed results, with some finding weak or even positive correlations between neighborhood opportunity and suicide.^25^, ^26^, ^27^ This suggests that the link between neighborhood opportunity and violent death may vary depending on the mode of injury.^26^

To address these gaps, this study uses high quality administrative data from the 2020 National Violent Death Reporting System (NVDRS) and the 2020 Decennial Census, linking these data with COI at the ZIP-code level. The underlying data include a full count of violent deaths and a full count of the U.S. child population in an area covering 83% of the total U.S. population. The authors analyze not only homicide mortality but also suicide, legal, and unintentional firearm deaths as well as deaths of undetermined manner, offering a comprehensive assessment of how neighborhood opportunity is associated with different types of violent death among children.

## METHODS

Table 1 lists the 44 component indicators of the COI, which are grouped into 14 subdomains and 3 domains.^9^ The component indicators are harmonized at the census block level and standardized using the z-score transformation. They are then combined into 14 subdomain composite z-scores that in turn form an overall index composite z-score. The composite z-scores are aggregated from the census block level to the census tract level and then further aggregated to ZIP codes using crosswalks published by the U.S. Department of Housing and Urban Development linking census tracts to ZIP codes.^28^ All U.S. ZIP codes are then ranked according to the overall index composite z-score and grouped into 5 levels (very low, low, moderate, high, and very high), each containing 20% of the national child population aged 0–19 years.

**Table 1:** Domains, Subdomains, and Indicators in the Child Opportunity Index 3.0

Education					
Early childhood education	Elementary education	Secondary and postsecondary education	Educational resources		
Private pre-K enrollment Public pre-K enrollment	Reading and math test scores Reading and math test score growth Poverty-adjusted reading and math test scores	Advanced placement course enrollment College enrollment in nearby institutions High-school graduation rate	Adult educational attainment Child enrichment-related nonprofits Teacher experience School poverty		
Health and environment					
Pollution	Healthy environments	Safety-related resources	Health resources		
Airborne microparticles Ozone concentration Industrial pollutants in air, water, or soil Hazardous waste dump sites	Fast food restaurant density Healthy food retailer density Extreme heat exposure NatureScore Walkability	Community safety-related non-profits Vacant housing	Health-related non-profits Health insurance coverage		
Social and economic					
Employment	Economic resources	Concentrated socioeconomic inequity	Housing resources	Social resources	Wealth
Employment rate High-skill employment rate Full-time year-round earnings	Median household income Poverty rate Public assistance rate	Adults with advanced degrees Very high-income households Adults without high-school degrees Very low-income households	Broadband access Crowded housing	Mobility-enhancing friendship networks Single-parent families Nonprofit organizations	Homeownership rate Aggregate home values Aggregate capital income Aggregate real estate taxes

*Notes:* This table was adapted from https://www.diversitydatakids.org/research-library/research-report/coi-30-technical-documentation.

Pre-K, prekindergarten.

The authors obtained counts of violent deaths for children aged 0–19 years linked to the decedents’ home residence ZIP code from the NVDRS. The NVDRS collects nationwide violent deaths data from law enforcement reports, medical examiner and coroner reports, and death certificates, and the ZIP codes of the victims’ resident addresses are available through a restricted data-use agreement.^29^ The NVDRS defines violent deaths as “death that results from the intentional use of physical force or power, threatened or actual, against oneself, another person, or a group or community.” For example, it includes not only firearm-related suicides but also suicides from suffocation, poisoning, and falls.^30^

The authors merged these data with ZIP Code Tabulation Area population counts of children aged 0–19 years from the 2020 Decennial Census and 2020 COI 3.0 ZIP code data. The authors selected 2020 data because the NVDRS has lower geographic coverage before 2020. Furthermore, 2020 provides high quality population counts from the Decennial Census, whereas analyses of intercensal years would require the use of sample-based population counts from the American Community Survey.

The 2020 NVDRS does not include data from Florida and Hawaii and omits some counties from Texas and California not reporting to the NVDRS.^31^ The final data set includes 61,494 violent deaths and 32,527 ZIP codes, and it covers 83% of the child population recorded in the 2020 Census. The omissions are slightly selective in terms of opportunity. The authors observe 16.9% of children residing in very low-opportunity areas rather than the expected 20% and slightly more than 20% of children at the other 4 opportunity levels. However, suicide and homicide death rates in the data set are practically identical to those reported by the Centers for Disease Control and Prevention for the same year and age group (Table 2): 3.3 homicide deaths per 100,000 children (CDC Wonder: 3.4) and 4.2 suicide deaths per 100,000 children (CDC Wonder: 4.3).^32^

**Table 2:** Crude Death Rates^a^ (Counts) Among Children Aged 0–19 Years by Cause of Death and Neighborhood Opportunity Level

Cause of death		Child Opportunity Index 3.0 opportunity levels					Very low to very high ratio^b^
	Total	Very low	Low	Moderate	High	Very high	
All deaths	8.42	15.27	9.63	7.26	5.72	3.76	4.06
	5,804	2,204	1,330	987	769	514	
Homicide	4.24	10.48	4.81	2.86	1.87	0.79	13.27
	2,926	1,512	665	389	252	108	
Suicide	3.33	3.33	3.79	3.60	3.22	2.69	1.24
	2,295	481	524	489	433	368	
Legal^c^	0.06	0.15	0.07	S	S	S	S
	43	21	10	S	S	S	
Unintentional firearm^d^	0.29	0.58	0.40	S	S	S	S
	203	84	55	S	S	S	
Undetermined	0.49	0.73	0.55	0.52	0.41	0.21	3.48
	337	106	76	71	55	29	

*Notes:* S denotes suppressed per NVDRS suppression rules.

a Deaths per 100,000 children aged 0-19 years.

b Ratio of crude death rates in very low- to very high-opportunity ZIP codes.

c Deaths caused by legal intervention (police or other authority).

d Unintentional firearm deaths that were self-inflicted, were inflicted by others, or had an unknown perpetrator. Child opportunity levels are nationally normed estimates that reflect 20% of the overall child population residing in each level of opportunity. NVDRS, National Violent Death Reporting System.

The authors computed crude child mortality rates by cause of death (c) across all ZIP codes with a given opportunity level (j) as 100,000 times d_cj_ divided by n_j_, where d_cj_ is the count of child deaths from a given cause c (all causes, suicide, homicide, legal intervention deaths, unintentional firearm, and deaths of undetermined manner) for each opportunity level j (very low, low, moderate, high, and very high), and n_j_ is the number of children at opportunity level j. The study was deemed exempt from further IRB review by Brandeis University (IRB Protocol Number 24161R-E).

## RESULTS

**Figure 1:**
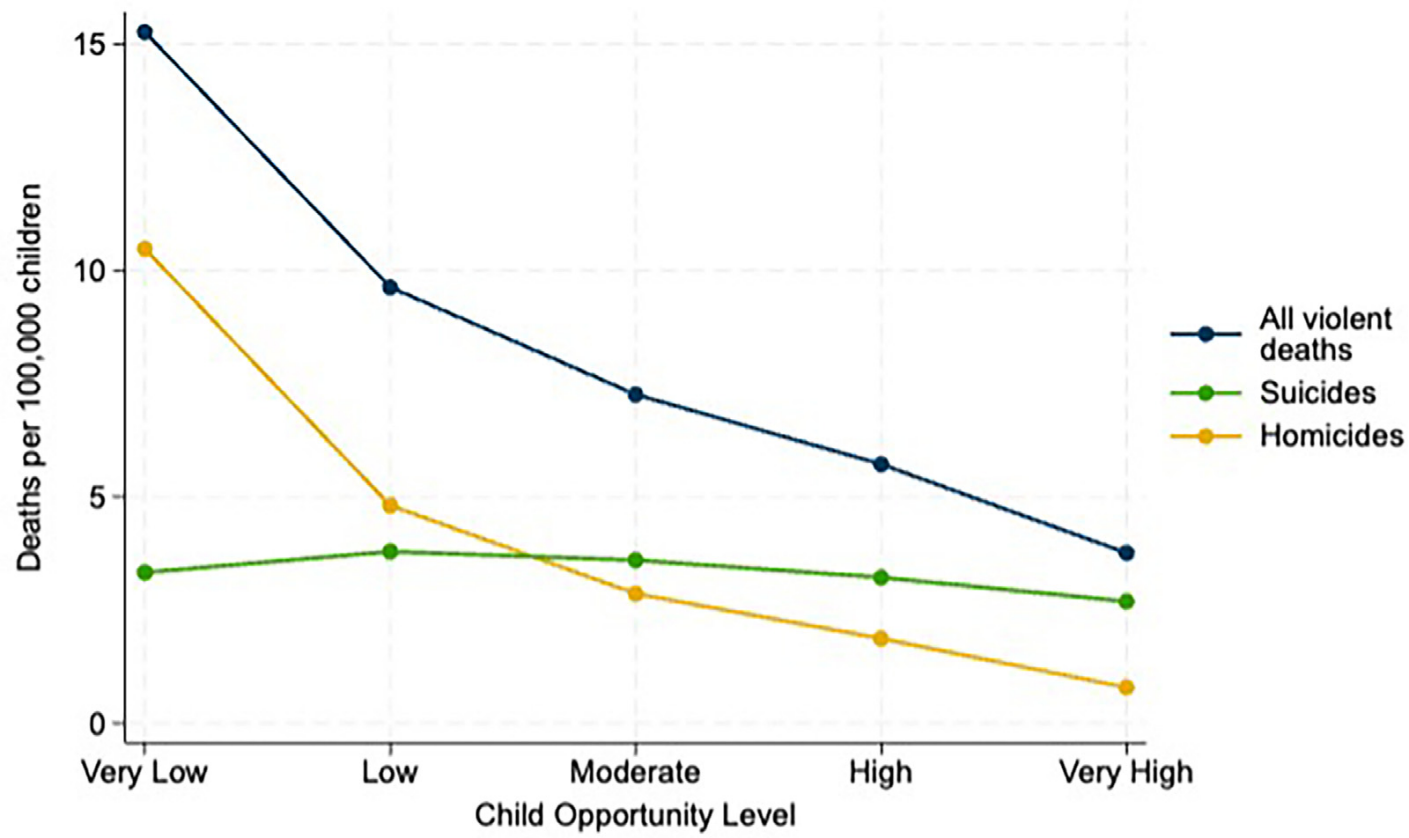
Crude death rates^a^ among children aged 0–19 years by cause of death and neighborhood opportunity level. *Notes:*^a^Deaths per 100,000 children aged 0–19 years. On the basis of statistics reported in Table 2, child opportunity levels were measured by the COI 3.0 for 2020. COI, Child Opportunity Index.

The crude violent death rate (all causes) declines monotonically (Figure 1) with neighborhood opportunity from 15.3 deaths per 100,000 children in very low-opportunity neighborhoods to 3.8 deaths per 100,000 children in very high-opportunity neighborhoods (Table 2). Homicide death rates are 13.3 times higher in very low- (10.5 deaths per 100,000 children) than in very high-opportunity neighborhoods (0.8 deaths per 100,000 children). In contrast, suicide mortality shows a weak, nearly flat, inverted U-shaped association with neighborhood opportunity, with 3.3 deaths per 100,000 children in very low- compared with 2.7 deaths per 100,000 children in very high-opportunity neighborhoods. Suicide mortality is highest in low-opportunity neighborhoods (3.8 deaths per 100,000 children).

Violent deaths from causes other than homicide or suicide are rare. Because of low cell counts (10 or fewer deaths), the authors are unable to report death rates for some causes and opportunity levels owing to NVDRS data suppression rules. Still, legal and unintentional firearm deaths show substantial disparities. Of the 43 deaths due to legal intervention, 72% (31 deaths) affected children residing in low- or very low-opportunity ZIP codes. One hundred and thirty-nine of the 203 unintentional firearm deaths (68%) occurred in low- or very low-opportunity neighborhoods. Violent deaths from undetermined causes also exhibit substantial disparities, with 54% (182 deaths) in very low- and low-opportunity neighborhoods.

## DISCUSSION

This study is the first to leverage COI 3.0 and 2020 NVDRS data to examine neighborhood disparities in children's violent death rates. Unlike prior research, which primarily examines homicide and firearm-related deaths in select metropolitan areas, this study investigates multiple causes of violent death—homicide, suicide, legal and unintentional firearm deaths, and deaths of undetermined manner. The underlying data include the full count of violent deaths as ascertained by the Centers for Disease Control and Prevention from nearly all U.S. states, covering an area home to 83% of the child population as of the 2020 Decennial Census. Therefore, it is less likely to suffer from geographic biases than previous studies that have focused on metropolitan areas or hospital data from predominantly urban areas.^33^^,^^34^

The study findings reveal stark disparities, with homicide mortality rates 13.3 times higher in very low- than in very high-opportunity neighborhoods. Deaths from other violent causes (legal, unintentional, and undetermined) also show steep opportunity gradients, with considerably elevated risks in very low- and low-opportunity neighborhoods compared with those in moderate- to very high-opportunity neighborhoods. However, neighborhood disparities in suicide rates follow a nearly flat, inverted U-shaped pattern, with highest rates observed in low-opportunity neighborhoods.

The findings align with those of previous studies that link neighborhood opportunity to elevated homicide and violent injury risks among children and contribute to the growing body of evidence that there are place-based differences in violent deaths and that these vary by cause of violent death. The study estimate for homicide death disparities between very low- and very high-opportunity neighborhoods is greater than those observed in studies using data from a single metropolitan area and is consistent with the very large disparities reported in a recent study using data from 35 children’s hospitals.^4^^,^^6^^,^^23^ The observed disparities in homicide mortality likely stem from the interplay of structural and social determinants of violence that operate at a neighborhood level. The COI provides a powerful measure to capture these multidimensional influences of children’s neighborhood contexts on their risk of violent death.^35^^,^^36^

Consistent with some previous studies, the analysis also shows that suicide mortality is only very weakly associated with low-neighborhood opportunity.^25^^,^^26^ The divergent patterns observed for homicide and suicide mortality may reflect differences in racial/ethnic composition of low- versus high-opportunity neighborhoods as well as racial/ethnic differences in suicide mortality risk. Using COI 3.0 data, Acevedo-Garcia et al.^37^ report that Black and Hispanic children are 7 times more likely than White children to reside in very low-opportunity neighborhoods.^37^ Stone and colleagues^38^ report that in 2020, suicide rates among White children were more than twice as high than among Black and Hispanic youth. The lower suicide risk among Black and Hispanic youth, who disproportionately reside in very low-opportunity neighborhoods, might attenuate and reduce what might otherwise be a stronger neighborhood opportunity gradient for White and Asian children. Further race-stratified analyses are warranted.

### Limitations

This study has several limitations. Because of the limited geographic coverage of the NVDRS, the analyses omit 17% of the 2020 child population. The data set has 3 percentage points fewer children in very low-opportunity areas than the national average. Furthermore, the reliance on ZIP codes may mask more localized variations in opportunity and death risks that may be better captured at the census tract level. These limitations may have slightly attenuated observed inequities in violent deaths across levels of neighborhood opportunity. COI data are available at the census tract level and should be used for spatial targeting of prevention efforts unless the spatial accuracy of ZIP code data for an intervention has been independently verified. Although the COI provides a multidimensional measure of neighborhood opportunity, it does not directly incorporate factors such as law enforcement presence, firearm accessibility, and access to mental health care, which may influence violent death risks. Furthermore, this study does not examine individual-level characteristics, which limits causal inference regarding the effects of neighborhood opportunity on violent deaths at the individual level.

## CONCLUSIONS

Despite these limitations, these findings validate the COI as a useful metric for identifying geographic disparities in children's risk of violent death, reinforcing its potential as a policy tool for targeting interventions.^35^^,^^36^ In addition, they emphasize the need for place-based violence prevention strategies that address structural conditions of neighborhood disadvantage rather than focusing solely on individual-level interventions. Finally, the results suggest that violence prevention strategies must differentiate between types of violent death. Specifically, suicide deaths exhibit a distinct pattern compared with other causes of death. Addressing the root causes of these disparities through multisectoral interventions is essential for advancing child health equity and reducing violence-related mortality in the most vulnerable communities.

## CRediT authorship contribution statement

**Manning Zhang:** Data curation, Formal analysis, Investigation, Software, Writing – original draft. **Clemens Noelke:** Writing – review & editing, Methodology, Resources, Software, Supervision, Funding acquisition, Validation. **Dolores Acevedo-Garcia:** Writing – review & editing, Funding acquisition. **Robert W. Ressler:** Writing – review & editing, Supervision, Methodology, Validation.
